# Racial/ethnic and gender disparity in the severity of NAFLD among people with diabetes or prediabetes

**DOI:** 10.3389/fphys.2023.1076730

**Published:** 2023-02-20

**Authors:** Magda Shaheen, Katrina M. Schrode, Marielle Tedlos, Deyu Pan, Sonia M. Najjar, Theodore C. Friedman

**Affiliations:** ^1^ Charles R. Drew University, Los Angeles, CA, United States; ^2^ Heritage College of Osteopathic Medicine, Ohio University, Athens, OH, United States

**Keywords:** NAFLD severity, prediabetes, diabetes, NHANES 2017–2018, race/ethnicity, gender

## Abstract

**Aim:** Non-alcoholic fatty liver disease (NAFLD) exhibits a racial disparity. We examined the prevalence and the association between race, gender, and NAFLD among prediabetes and diabetes populations among adults in the United States.

**Methods:** We analyzed data for 3,190 individuals ≥18 years old from the National Health and Nutrition Examination Survey (NHANES) 2017–2018. NAFLD was diagnosed by FibroScan^®^ using controlled attenuation parameter (CAP) values: S0 (none) < 238, S1 (mild) = 238–259, S2 (moderate) = 260–290, S3 (severe) > 290. Data were analyzed using Chi-square test and multinomial logistic regression, adjusting for confounding variables and considering the design and sample weights.

**Results:** Of the 3,190 subjects, the prevalence of NAFLD was 82.6%, 56.4%, and 30.5% (*p* < 0.0001) among diabetes, prediabetes and normoglycemia populations respectively. Mexican American males with prediabetes or diabetes had the highest prevalence of severe NAFLD relative to other racial/ethnic groups (*p* < 0.05). In the adjusted model, among the total, prediabetes, and diabetes populations, a one unit increase in HbA1c was associated with higher odds of severe NAFLD [adjusted odds ratio (AOR) = 1.8, 95% confidence level (CI) = 1.4–2.3, *p* < 0.0001; AOR = 2.2, 95% CI = 1.1–4.4, *p* = 0.033; and AOR = 1.5, 95% CI = 1.1–1.9, *p* = 0.003 respectively].

**Conclusion:** We found that prediabetes and diabetes populations had a high prevalence and higher odds of NAFLD relative to the normoglycemic population and HbA1c is an independent predictor of NAFLD severity in prediabetes and diabetes populations. Healthcare providers should screen prediabetes and diabetes populations for early detection of NAFLD and initiate treatments including lifestyle modification to prevent the progression to non-alcoholic steatohepatitis or liver cancer.

## 1 Introduction

Non-alcoholic fatty liver disease (NAFLD) is a liver condition frequently associated with obesity (Friedman et al., 2018). The prevalence of NAFLD in the United States has been increasing over time from 18% in 1988–1991 to be between 25% to over 50% currently based on transient elastography (Kim et al., 2020; Zhang et al., 2021; Noureddin et al., 2022). NAFLD can be moderate or severe and can progress to non-alcoholic steatohepatitis (NASH), fibrosis, cirrhosis and liver cancer. We recently reported that Mexican Americans, but not other Hispanics have a high prevalence of hepatic steatosis compared to non-Hispanic Whites (Shaheen et al., 2021a), and that male Mexican Americans were at the highest risk for NAFLD (Shaheen et al., 2021b).

Over 70% of patients with diabetes in the United States have NAFLD (Xia et al., 2019). NAFLD and diabetes have a complex bidirectional relationship where NAFLD increases the risk of developing type 2 diabetes (T2D) up to 5-fold, and having T2D can promote the progression of mild hepatic steatosis to a more severe state (Xia et al., 2019).

Prediabetes is defined by a hemoglobin A1c (HbA1c) between 5.7% and 6.4%, fasting plasma glucose (FPG) between 100–125 mg/dL, or a glucose of 140–199 mg/dL 2 h after an oral glucose tolerance test. Prediabetes is a significant risk factor for T2D and macro-vascular diseases such as diastolic heart failure and coronary heart disease (Mai et al., 2021), and it often coexists with hepatic steatosis (Rajput and Ahlawat, 2019). According to the CDC, approximately 96 million adults in the United States have prediabetes with 80% being unaware of their condition (CDC, 2021). With the DPP study showing intensive lifestyle intervention can delay the progression from prediabetes to diabetes (Knowler et al., 2002), there is controversy on whether or not prediabetes should be treated pharmacologically (Shaw, 2019).

Our goal was to examine the prevalence of NAFLD severity by prediabetes status. In addition, we examined the independent association of NAFLD severity and HbA1c level in subjects with prediabetes as well as diabetes in a representative sample of the United States non-institutionalized adult population =>18 years. We also examined the independent association of race/ethnicity and gender and NAFLD severity among subjects with prediabetes and subjects with overt diabetes.

## 2 Research design and methods

### 2.1 Study population

We analyzed data of 3,190 participants =>18 years old from the National Health and Nutrition Examination Survey (NHANES) 2017–2018. NHANES samples the non-institutionalized population in the United States using a complex, multistage probability sample. NHANES protocols were approved by the National Center for Health Statistics Research Ethics Review Board. Our analysis of these publicly available data was exempt from Charles R. Drew University IRB review. Our analysis consisted of NHANES participants who were examined for hepatic steatosis using ultrasound and vibration controlled transient elastography (VCTE).

### 2.2 Dependent variable

Hepatic steatosis was assessed using FibroScan^®^ which uses ultrasound and VCTE to measure the controlled attenuation parameter (CAP). Hepatic steatosis was categorized based on the median CAP dB/m for steatosis grades into S0 (no steatosis) < 238; S1 (mild steatosis) = 238–259; S2 (moderate steatosis) = 260–290; and S3 (severe steatosis) > 290 (MSKC Center, 2018). For classification of NAFLD, subjects were considered to have NAFLD if they had hepatic steatosis and did not have any exclusion criteria [transferrin level >50%, hepatitis B, hepatitis C, excessive alcohol use (i.e., an average of more than two drinks/day for men or one drink/day for women), or prescription medications that might cause hepatic steatosis (i.e., corticosteroids, antiarrhythmics, anticancer-antimetabolites, anticancer-hormonal drugs, anti-convulsant drugs, or nucleoside/nucleotide reverse transcriptase inhibitors)] (Kim et al., 2022).

### 2.3 Independent variables

Participants were considered normoglycemic if they had HbA1c <5.7% and FPG <100 mg/dL; considered to have prediabetes if their HbA1c was 5.7%–6.4%, FPG 100–125 mg/dL, or they self-reported prediabetes; and to have diabetes if their HbA1c was ≥6.5, FPG ≥126, or they self-reported diabetes. Patients with self-reported diabetes and an HbA1c between 5.7% and 6.4% or FPG 100–125 mg/dL were categorized as having diabetes, not prediabetes. Among those with diabetes or prediabetes, we also investigated prescribed medication as a categorical variable where possible treatments were hypoglycemic pills, insulin, or both, or not taking medication. Other variables included in the analysis were: demographics (age, gender, race/ethnicity, education, language spoken, and income ratio), behavioral variables [physical activity status, smoking status, alcohol consumption, and diet quality using healthy eating index (HEI)], body composition (waist-hip ratio and body mass index), laboratory values [cholesterol, HDL, triglyceride, hemoglobin A1c (HbA1c), highly-sensitive C-reactive protein (hsCRP), AST and ALT, and comorbidity (hypertension)].

For most categorical variables, the categories used were those defined by NHANES. Physical activity was categorized into three categories based on whether reported activity met national guidelines. Participants were categorized as current, never, and former drinkers based on their responses to questions about frequency and quantity of alcohol consumption. We also calculated the average daily drinking volume by multiplying the number of drinks by the number of drinking days. The quantity can then be used to find the average daily drinking volume by dividing the quantity by 365, the total number of days in a year. We included individuals who never drank alcohol and those who are light drinkers in our analysis. Laboratory variables were categorized according to standard clinical guidelines.

### 2.4 Statistical analyses

Descriptive statistics were used to depict the population characteristics. Categorical data were presented as unweighted number and weighted percent. Continuous variables were presented as mean and standard error. Missing data was <8% for the variables. We used chi square test to examine the statistical difference in the prevalence of NAFLD as well as differences in the population characteristics by the diabetes/prediabetes status. We used multinomial regression to examine the association between NAFLD severity and diabetes/prediabetes status adjusting for the population characteristics and laboratory variables. The confounding variables were age, gender, smoking status, HEI, alcohol use, BMI values, waist-to-hip ratio, physical activity, total cholesterol, triglyceride values, hsCRP, ALT and AST levels, and hypertension. In addition, we used multinomial regression to examine the independent association between NAFLD severity and HbA1c level. We repeated the multinomial logistic regression among the participants with prediabetes and diabetes.

We also analyzed the data using hierarchical block logistic regression to determine the independent variables that explain the statistically significant amount of variance in NAFLD after accounting for all the other independent variables. In this analysis, we ran the model several times, first including demographic variables, then adding behavioral variables, followed by body composition variables, laboratory variables, and finally comorbidity. Results were presented as adjusted odds ratio (AOR) and 95% confidence interval (CI) and *p*-value of <0.05 was considered statistically significant. Data were analyzed using SAS v9.4 including the design, and the sample weights provided by the NCHS that were used to correct for differential selection probabilities and to adjust for non-coverage and non-response.

## 3 Results

### 3.1 Population characteristics of the total population

Table 1 shows the population characteristics and the prevalence of NAFLD severity by diabetes/prediabetes status. Of the 3,190 subjects in our sample, 18.4% were 65 years and older, 10.0% were Black, 8.4% were Mexican Americans, and 6.0% were other Hispanics (Table 1). About half of the population were male (48.5%), 9.8% had less than high-school education, 31.4% had at least a college degree, and 12.2% had an income below the poverty line (FIR <1). Most of the participants (84.0%) spoke English at home, 16.2% were current smokers, 76.4% currently drank alcohol, 18.6% were physically-inactive, 71.3% had a poor diet, 53.6% had high waist-to-hip ratio (≥0.85 for females, ≥1.0 for males) and 41.4% were obese by BMI. In terms of laboratory tests, 10.3% had high total cholesterol, 15.7% had low HDL, 16.8% had high levels of triglycerides, 33.0% had significant inflammation as indicated by hsCRP level, 4.0% had abnormal AST levels, 3.6% had abnormal ALT levels, and 36.6% had hypertension (Table 1). According to our classification, 40.5% had prediabetes, and 13.4% had diabetes (Table 1).

**TABLE 1:** Population characteristics and prevalence of NAFLD severity by diabetes/prediabetes status.

	Overall	Normoglycemic	Prediabetes	Diabetes	*p*-value
		glucose <100 mg/dL & A1c <5.7%	glucose 100–125 mg/dL or A1c 5.7%–6.4% or self-report	glucose 126 mg/dL or A1c 6.5% or self-report	
	n = 3,190	n = 1,317 (46.0%)	n = 1,277 (40.5%)	n = 596 (13.4%)	
NAFLD					<.0001
no/mild	1,612 (52.0)	899 (69.5)	573 (43.6)	140 (17.3)	
Moderate	524 (16.2)	174 (13.0)	250 (20.0)	100 (15.8)	
Severe	1,054 (31.8)	244 (17.5)	454 (36.4)	356 (66.8)	
Race/ethnicity					0.3995
Mexican American	422 (8.4)	174 (46.2)	161 (39.5)	87 (14.3)	
Other Hispanic	273 (6.0)	95 (44.8)	122 (43.2)	56 (12.0)	
Non-Hispanic White	1,193 (65.8)	537 (47.0)	462 (40.2)	194 (12.8)	
Non-Hispanic Black	701 (10.0)	264 (45.1)	288 (38.2)	149 (16.8)	
Other Race	601 (9.9)	247 (41.0)	244 (44.3)	110 (14.7)	
Gender					0.0163
Male	1,556 (48.5)	582 (41.3)	650 (44.5)	324 (14.2)	
Female	1,634 (51.5)	735 (50.4)	627 (36.8)	272 (12.7)	
Age					<.0001
18–19	156 (3.2)	122 (78.4)	33 (21.3)	1 (0.3)	
20–34	712 (27.2)	476 (65.4)	213 (32.4)	23 (2.2)	
35–49	698 (23.2)	316 (49.2)	296 (41.1)	86 (9.7)	
50–64	904 (28.0)	258 (35.5)	409 (45.6)	237 (18.9)	
65+	720 (18.4)	145 (23.9)	326 (47.5)	249 (28.6)	
Education level					0.0737
Less than high school	546 (9.8)	199 (41.0)	211 (39.7)	136 (19.4)	
High school	793 (28.0)	346 (46.7)	301 (37.9)	146 (15.3)	
Some college	1,076 (30.8)	457 (46.0)	425 (40.4)	194 (13.6)	
At least college degree	775 (31.4)	315 (47.0)	340 (43.3)	120 (9.7)	
Language spoken at home					0.1797
English	2,382 (84.0)	1,000 (46.4)	954 (40.6)	428 (13.1)	
Spanish	206 (3.4)	57 (34.0)	91 (46.1)	58 (19.9)	
Both English and Spanish	348 (7.5)	155 (51.2)	129 (36.0)	64 (12.7)	
Other	254 (5.2)	105 (40.9)	103 (43.1)	46 (15.9)	
Federal Income ratio (FIR)					0.0894
<1	581 (12.2)	273 (54.3)	208 (33.5)	100 (12.2)	
1–2	868 (19.7)	335 (45.5)	354 (40.0)	179 (14.5)	
>2	1741 (68.2)	709 (44.7)	715 (42.0)	317 (13.3)	
Waist-hip ratio					<.0001
Healthy	1,399 (46.4)	734 (57.4)	534 (37.6)	131 (4.9)	
risk for women ( 0.85)/risk for men (>1.0)	1791 (53.6)	583 (36.2)	743 (43.1)	465 (20.8)	
BMI					<.0001
Normal	902 (28.0)	527 (64.8)	292 (30.7)	83 (4.6)	
Overweight	1,009 (30.6)	400 (43.6)	438 (45.9)	171 (10.4)	
Obese	1,279 (41.4)	390 (35.1)	547 (43.2)	342 (21.6)	
Alcohol consumption					0.0001
Current	2,225 (76.4)	993 (48.3)	881 (40.3)	351 (11.4)	
Former	635 (16.1)	194 (35.5)	258 (43.2)	183 (21.3)	
Never	330 (7.5)	130 (45.2)	138 (37.4)	62 (17.4)	
Smoking status					0.0002
Current	549 (16.2)	241 (49.2)	234 (40.2)	74 (10.6)	
Former	723 (23.8)	225 (37.4)	301 (44.2)	197 (18.4)	
Never	1918 (60.0)	851 (48.6)	742 (39.2)	325 (12.2)	
Physical activity					<.0001
Inactive	714 (18.6)	220 (30.6)	300 (48.1)	194 (21.3)	
Does not meet guidelines	497 (15.1)	171 (39.9)	204 (40.3)	122 (19.8)	
Meets guidelines	1979 (66.3)	926 (51.7)	773 (38.5)	280 (9.8)	
Healthy eating index					0.1566
poor diet	2,234 (71.3)	946 (45.4)	910 (41.9)	378 (12.7)	
needs improvement	856 (26.1)	328 (46.5)	335 (38.0)	193 (15.5)	
good diet	100 (2.6)	43 (58.8)	32 (29.1)	25 (12.0)	
Cholesterol					0.0002
good (<200 mg/dL)	2069 (62.7)	895 (48.6)	752 (36.7)	422 (14.6)	
elevated (200–239 mg/dL)	799 (27.0)	322 (43.2)	368 (46.6)	109 (10.2)	
high (240 mg/dL)	322 (10.3)	100 (37.6)	157 (47.8)	65 (14.6)	
HDL					<.0001
low (<40 mg/dL)	547 (15.7)	172 (33.4)	207 (37.0)	168 (29.6)	
borderline risk (40–59 mg/dL)	1729 (54.6)	678 (43.2)	714 (43.6)	337 (13.2)	
healthy (60 mg/dL)	914 (29.8)	467 (57.9)	356 (36.7)	91 (5.4)	
Triglycerides					<.0001
normal (<150 mg/dL)	2,160 (67.3)	1,006 (52.1)	852 (39.3)	302 (8.6)	
borderline (150–199 mg/dL)	493 (15.9)	152 (36.4)	208 (44.7)	133 (18.9)	
high (200 mg/dL)	537 (16.8)	159 (30.8)	217 (41.7)	161 (27.5)	
CRP					<.0001
normal (0.1- <1 mg/dL)	971 (31.3)	510 (56.5)	350 (36.6)	111 (6.9)	
mild inflammation (1-<3 mg/dL)	1,128 (35.8)	462 (47.1)	458 (40.1)	208 (12.8)	
significant inflammation (3-<10 mg/dL)	865 (26.2)	284 (36.4)	379 (44.6)	202 (19.1)	
high significant inflammation 10 mg	226 (6.8)	61 (29.4)	90 (45.1)	75 (25.5)	
AST					0.5827
normal (<40 U/L)	3,066 (96.0)	1,266 (45.9)	1,233 (40.8)	567 (13.4)	
elevated (>40 U/L)	124 (4.0)	51 (49.8)	44 (35.0)	29 (15.2)	
ALT					0.7668
normal (<56 U/L)	3,081 (96.4)	1,275 (46.0)	1,238 (40.7)	568 (13.3)	
elevated (>56 U/L)	109 (3.6)	42 (47.4)	39 (36.4)	28 (16.2)	
HOMA*					<.0001
healthy (<2.0)	558 (41.0)	285 (52.6)	235 (45.0)	38 (2.5)	
borderline insulin resistance (2.0–2.9)	326 (20.2)	94 (30.7)	196 (61.4)	36 (8.0)	
insulin resistance (3+)	670 (38.8)	71 (10.5)	350 (59.7)	249 (29.8)	
Hypertension					<.0001
Yes	1,354 (36.6)	353 (28.8)	585 (45.5)	416 (25.7)	
No	1836 (63.4)	964 (56.0)	692 (37.7)	180 (6.3)	
					
	mean ± sem	mean ± sem	mean ± sem	mean ± sem	
HbA1c	5.64 ± 0.02	5.23 ± 0.01	5.60 ± 0.02	7.20 ± 0.08	<.0001
Fasting plasma glucose (mg/dL)*	109.8 ± 1.22	93.4 ± 0.23	105.9 ± 0.36	160.4 ± 5.22	<.0001
*reduced sample					

### 3.2 Population characteristics by diabetes/prediabetes status

Higher prevalence of diabetes and prediabetes were found among those who were male, age 50 years and older, with high-risk waist-to-hip ratio, who were overweight or obese, former smokers, former alcoholics, those who were physically inactive, with high cholesterol, with low HDL, high triglycerides, high hsCRP, those with insulin resistance and those with hypertension compared to the reference groups (*p* < 0.05) (Table 1). There was no racial/ethnic difference in the prevalence of normoglycemic, diabetes, and prediabetes (*p* > 0.05) (Table 1, Supplementary Table S1). About 65% of participants with diabetes were taking some kind of antiglycemic medication to manage their condition, while only 1.5% of participants with prediabetes were taking any prescription antiglycemic medication (Supplementary Table S2A).

### 3.3 Prevalence of NAFLD severity among the prediabetes and diabetes populations

Overall, the prevalence of moderate NAFLD was 16.2% and that of severe NAFLD was 31.8%. The prevalence of moderate NAFLD did not vary greatly between groups, with 13% among the normoglycemic population, 20% in those with prediabetes, and 15.8% in those with diabetes. Prevalence of severe NAFLD was 17.5% in the normoglycemic group, 36.4% among those with prediabetes, and 66.8% in those with diabetes (*p* < 0.0001) (Table 1). Among those with prediabetes, the prevalence of severe NAFLD was 71.6% in those being treated with antiglycemic medications compared to 35.8% in those who were not (*p* < 0.0001) (Supplementary Table S2A). There were no significant differences by treatment among those with diabetes.

### 3.4 Racial/ethnic and gender difference in the prevalence of NAFLD severity by diabetes/prediabetes status

**FIGURE 1:**
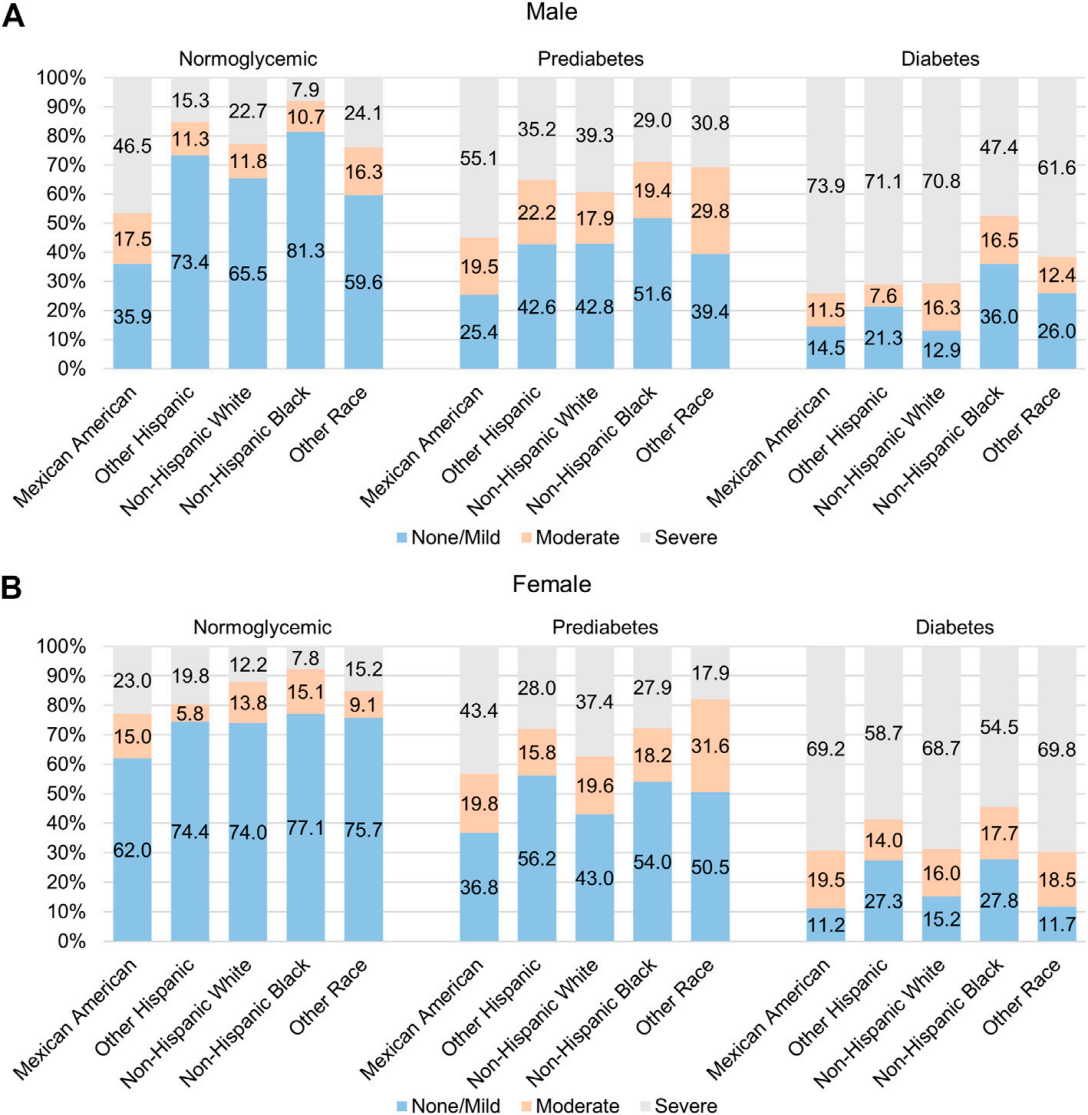
Racial/ethnic variation in prevalence of NAFLD among those with diabetes and pre-diabetes. Prevalence of moderate and severe NAFLD among **(A)** males and **(B)** females of different racial/ethnic groups who are normoglycemic (left) or have pre-diabetes (middle) or diabetes (right).

Among males, Mexican Americans had the highest prevalence of severe NAFLD (46.5% in normoglycemic, 55.1% in prediabetes, and 73.9% in the diabetes population) relative to the other racial/ethnic groups, but this disparity was only significant in the normoglycemic and diabetic populations (*p* < 0.05; Figure 1, Supplementary Table S1). Among females, Mexican Americans had the highest prevalence of severe NAFLD in all groups (23.0% in normoglycemic, 43.4% in prediabetes, and 69.2% in the diabetes population) compared to the other racial/ethnic groups, but the difference was only significant in the normoglycemic individuals (*p* < 0.05; Figure 1; Supplementary Table S1).

### 3.5 Multivariable analysis

Table 2 shows the multinomial logistic regression of the association between NAFLD severity and HbA1c level and other correlates in the total population, and separately in the populations with prediabetes and diabetes.

**TABLE 2:** Adjusted odds ratio (AOR) and 95% confidence interval for factors associated with NAFLD severity (Reference population of normal/mild) in the total population, and in diabetes and pre-diabetes populations from NHANES 2017–18 (*n* = 3,190).

N = 3,190	Overall		Normoglycemic		Prediabetes		Diabetes	
	moderate	severe	moderate	severe	moderate	severe	moderate	severe
	AOR	AOR	AOR	AOR	AOR	AOR	AOR	AOR
**HbA1c**	1.2 [ 0.9–1.5]	**1.8 [ 1.4–2.3]**	1.1 [ 0.4–3.0]	0.8 [ 0.3–1.9]	0.6 [ 0.3–1.2]	**2.2 [ 1.1–4.4]**	1.1 [ 0.8–1.4]	**1.5 [ 1.1–1.9]**
**Race/ethnicity**								
Mexican American vs. Non-Hispanic White	**1.9 [ 1.2–3.2]**	**2.6 [ 1.3–4.9]**	**3.1 [ 1.4–6.9]**	**3.0 [ 1.1–8.1]**	1.4 [ 0.4–4.3]	2.0 [ 0.8–5.0]	2.3 [ 0.2–30.7]	3.4 [ 0.8–15.2]
Non-Hispanic Black vs. Non-Hispanic White	0.9 [ 0.6–1.4]	**0.5 [ 0.4–0.7]**	1.2 [ 0.8–1.8]	**0.4 [ 0.3–0.6]**	0.9 [ 0.4–2.0]	0.5 [ 0.3–1.0]	0.4 [ 0.1–1.2]	**0.3 [ 0.1–0.8]**
Other Hispanic vs. Non-Hispanic White	0.8 [ 0.6–1.3]	0.8 [ 0.5–1.2]	0.8 [ 0.2–3.5]	1.0 [ 0.2–4.1]	0.7 [ 0.3–2.1]	0.5 [ 0.2–1.5]	0.3 [ 0.0–6.5]	0.8 [ 0.2–3.4]
Other Race vs. Non-Hispanic White	**1.8 [ 1.0–3.1]**	1.2 [ 0.7–2.0]	1.8 [ 0.9–3.4]	**2.5 [ 1.1–5.7]**	1.7 [ 0.7–3.7]	0.6 [ 0.3–1.2]	1.3 [ 0.5–3.8]	1.5 [ 0.5–4.1]
**Gender**								
female vs. male	0.7 [ 0.5–1.1]	**0.5 [ 0.3–0.8]**	0.6 [ 0.3–1.1]	**0.4 [ 0.2–0.7]**	0.9 [ 0.6–1.5]	0.6 [ 0.2–1.3]	1.6 [ 0.5–4.7]	1.2 [ 0.5–3.1]
**Age**								
18–19 vs. 20–34	0.6 [ 0.3–1.3]	0.7 [ 0.4–1.4]	0.5 [ 0.1–1.6]	0.9 [ 0.6–1.4]	0.7 [ 0.1–3.9]	0.9 [ 0.2–4.1]		3.8 [ 0.2–83.6]
35–49 vs. 20–34	**1.4 [ 1.0–2.1]**	1.2 [ 0.8–1.7]	1.4 [ 0.9–2.3]	0.9 [ 0.4–1.9]	1.6 [ 0.9–3.0]	1.3 [ 0.5–3.2]	4.6 [ 0.5–39.9]	**10.2 [ 2.2–47.3]**
50–64 vs. 20–34	**2.4 [ 1.3–4.5]**	**1.7 [ 1.2–2.6]**	1.6 [ 0.7–3.8]	1.5 [ 0.9–2.6]	**3.0 [ 1.1–7.7]**	1.6 [ 0.6–4.5]	**22.8 [ 1.5–346]**	**31.2 [ 4.7–209]**
65 + vs. 20–34	**2.1 [ 1.1–4.1]**	1.4 [ 0.8–2.4]	1.5 [ 0.4–4.9]	1.1 [ 0.3–4.0]	**3.2 [ 1.4–7.4]**	1.7 [ 0.8–3.4]	6.1 [ 0.4–94.0]	**6.9 [ 0.8–58.9]**
**Education level**								
Less than high school vs. High school	1.4 [ 1.0–2.0]	0.7 [ 0.4–1.2]	0.4 [ 0.1–1.3]	0.7 [ 0.4–1.5]	1.6 [ 0.9–3.0]	0.7 [ 0.4–1.2]	**5.5 [ 2.0–14.8]**	0.8 [ 0.3–2.4]
Some college vs. High school	1.2 [ 0.7–2.0]	1.1 [ 0.7–1.9]	1.1 [ 0.5–2.4]	0.9 [ 0.4–2.1]	0.8 [ 0.4–1.6]	1.2 [ 0.6–2.3]	**7.2 [ 3.3–15.8]**	2.1 [ 0.9–4.6]
At least college degree vs. High school	1.4 [ 0.7–2.7]	0.9 [ 0.5–1.8]	1.3 [ 0.5–3.4]	0.8 [ 0.3–2.4]	1.1 [ 0.5–2.3]	1.1 [ 0.6–1.9]	2.3 [ 0.8–6.9]	0.8 [ 0.3–2.5]
**Language spoken at home**								
Spanish vs. English	0.8 [ 0.4–1.4]	0.7 [ 0.4–1.4]	1.6 [ 0.4–6.7]	1.4 [ 0.3–7.6]	0.7 [ 0.2–2.3]	1.0 [ 0.4–2.3]	0.1 [ 0.0–2.3]	**0.1 [ 0.0–0.4]**
Both English and Spanish vs. English	0.8 [ 0.4–1.4]	1.0 [ 0.5–2.0]	0.6 [ 0.1–2.6]	1.1 [ 0.3–3.7]	1.1 [ 0.4–3.0]	1.0 [ 0.3–3.8]	0.8 [ 0.0–14.9]	0.9 [ 0.2–4.8]
Other vs. English	1.2 [ 0.6–2.4]	1.5 [ 0.6–3.5]	0.5 [ 0.2–1.3]	0.8 [ 0.3–2.2]	1.6 [ 0.5–4.8]	1.6 [ 0.5–5.1]	1.0 [ 0.2–4.5]	2.4 [ 0.4–14.4]
**Income ratio**								
<1 vs. > 2	0.8 [ 0.6–1.2]	0.8 [ 0.6–1.1]	0.6 [ 0.3–1.2]	1.0 [ 0.6–1.9]	0.7 [ 0.4–1.4]	0.7 [ 0.4–1.2]	2.4 [ 0.7–7.6]	0.9 [ 0.3–2.2]
1–2 vs. > 2	1.1 [ 0.7–1.8]	0.9 [ 0.7–1.2]	0.7 [ 0.3–1.6]	0.9 [ 0.5–1.8]	1.3 [ 0.7–2.3]	1.0 [ 0.7–1.6]	1.3 [ 0.6–2.9]	0.6 [ 0.3–1.4]
**Waist-hip ratio**								
risk for women (≥0.85)/risk for men (≥1.0) vs. healthy	**2.1 [ 1.3–3.4]**	**2.4 [ 1.6–3.4]**	**3.1 [ 1.5–6.6]**	**5.1 [ 2.8–9.3]**	1.3 [ 0.7–2.4]	1.3 [ 0.8–2.1]	1.5 [ 0.4–5.9]	1.7 [ 0.6–4.9]
**BMI**								
overweight vs. normal	**3.8 [ 2.3–6.4]**	**6.8 [ 3.9–11.7]**	**2.4 [ 1.3–4.6]**	**5.0 [ 1.6–15.3]**	**5.2 [ 2.8–9.7]**	**7.8 [ 3.6–16.6]**	**6.0 [ 1.5–23.5]**	**12.4 [ 4.2–36.3]**
obese vs. normal	**6.4 [ 3.7–11.1]**	**22.9 [11.8–44.4]**	**3.7 [ 1.9–7.1]**	**20.2 [ 6.6–61.8]**	**13.3 [ 6.0–29.4]**	**30.6 [13.7–68.7]**	4.5 [ 0.8–25.5]	**36.1 [ 8.4–154]**
**Smoking status**								
Current vs. Never	0.8 [ 0.5–1.3]	0.7 [ 0.5–1.1]	0.9 [ 0.5–1.7]	1.1 [ 0.5–2.2]	1.2 [ 0.5–2.8]	0.6 [ 0.3–1.1]	**0.2 [ 0.1–0.5]**	**0.3 [ 0.1–0.7]**
Former vs. Never	1.1 [ 0.8–1.5]	1.0 [ 0.7–1.5]	1.0 [ 0.5–2.2]	1.5 [ 0.7–3.2]	1.3 [ 0.6–2.4]	0.9 [ 0.5–1.6]	0.5 [ 0.1–2.4]	1.0 [ 0.4–2.7]
**Alcohol consumption**								
current drinker vs. never drank	1.5 [ 1.0–2.4]	0.6 [ 0.3–1.2]	1.7 [ 0.8–3.6]	0.8 [ 0.4–1.5]	1.0 [ 0.4–2.1]	**0.4 [ 0.2–1.0]**	2.4 [ 0.7–8.7]	0.7 [ 0.3–2.0]
former drinker vs. never drank	1.5 [ 0.9–2.6]	0.5 [ 0.3–1.0]	1.5 [ 0.6–3.9]	0.7 [ 0.3–2.0]	1.0 [ 0.5–2.1]	**0.3 [ 0.1–0.9]**	2.3 [ 0.8–6.4]	0.8 [ 0.2–2.7]
**Physical activity**								
Inactive vs. Meets guidelines	1.1 [ 0.8–1.5]	1.3 [ 0.9–1.9]	2.1 [ 0.9–4.8]	1.7 [ 0.9–3.1]	0.6 [ 0.3–1.2]	0.9 [ 0.6–1.3]	0.7 [ 0.3–1.6]	1.3 [ 0.7–2.8]
Does not meet guidelines vs. Meets guidelines	0.9 [ 0.5–1.6]	1.6 [ 1.0–2.7]	1.5 [ 0.6–3.7]	2.0 [ 1.0–4.1]	**0.5 [ 0.3–0.9]**	1.2 [ 0.6–2.5]	1.5 [ 0.5–4.8]	1.4 [ 0.7–2.9]
**Healthy eating index**								
poor diet vs. good diet	1.7 [ 0.7–4.1]	1.0 [ 0.2–3.8]	0.8 [ 0.3–2.7]	0.4 [ 0.1–2.2]	3.5 [ 0.9–13.6]	6.1 [ 0.8–47.8]	1.4 [ 0.1–17.9]	0.5 [ 0.0–7.5]
needs improvement vs. good diet	1.3 [ 0.5–3.3]	0.9 [ 0.3–3.3]	0.6 [ 0.2–2.1]	0.4 [ 0.1–2.2]	2.7 [ 0.6–12.2]	3.7 [ 0.5–26.8]	1.3 [ 0.1–15.4]	0.9 [ 0.1–10.9]
**Cholesterol**								
elevated (200–239 mg/dL) vs. good (<200 mg/dL)	0.8 [ 0.5–1.2]	1.0 [ 0.6–1.4]	0.9 [ 0.5–1.6]	0.7 [ 0.4–1.2]	0.8 [ 0.5–1.3]	1.1 [ 0.7–1.9]	0.9 [ 0.2–4.4]	1.5 [ 0.5–4.9]
high (≥240 mg/dL) vs. good (<200 mg/dL)	0.8 [ 0.6–1.1]	0.8 [ 0.6–1.2]	0.6 [ 0.3–1.2]	0.9 [ 0.4–1.7]	1.0 [ 0.4–2.4]	0.8 [ 0.4–1.6]	**0.2 [ 0.0–0.7]**	**0.2 [ 0.1–0.6]**
**HDL**								
low (<40 mg/dL) vs. healthy (≥60 mg/dL)	1.1 [ 0.5–2.4]	**2.0 [ 1.1–3.6]**	1.1 [ 0.4–2.8]	**3.5 [ 1.5–8.6]**	0.9 [ 0.4–2.2]	1.0 [ 0.4–2.1]	3.1 [ 0.4–21.8]	**4.4 [ 1.3–15.5]**
borderline risk (40–59 mg/dL) vs. healthy (≥60 mg/dL)	1.1 [ 0.7–1.6]	1.5 [ 1.0–2.3]	0.8 [ 0.4–1.5]	**1.9 [ 1.2–3.3]**	0.9 [ 0.5–1.7]	0.8 [ 0.5–1.4]	**3.7 [ 1.3–10.7]**	**4.2 [ 1.5–11.8]**
**Triglycerides**								
borderline (150–199 mg/dL) vs. normal (<150 mg/dL)	**1.7 [ 1.3–2.4]**	**1.7 [ 1.1–2.8]**	**2.7 [ 1.4–5.0]**	1.4 [ 0.6–3.0]	1.3 [ 0.8–2.2]	**2.2 [ 1.4–3.5]**	2.5 [ 0.8–7.4]	2.5 [ 0.7–8.9]
high (≥200 mg/dL) vs. normal (<150 mg/dL)	**1.7 [ 1.2–2.4]**	**2.8 [ 1.9–4.1]**	2.1 [ 0.9–4.7]	1.9 [ 0.8–4.6]	1.5 [ 0.8–2.7]	**3.9 [ 2.0–7.7]**	2.2 [ 0.8–6.0]	**3.8 [ 1.5–9.4]**
**CRP**								
mild inflammation (1-<3 mg/dL) vs. normal (0.1- <1 mg/dL)	1.4 [ 0.9–2.2]	1.2 [ 0.7–2.0]	1.4 [ 0.9–2.2]	1.0 [ 0.5–2.0]	1.6 [ 0.8–3.0]	1.6 [ 0.8–3.3]	**3.0 [ 1.1–8.5]**	1.5 [ 0.8–3.0]
significant inflammation (3-<10 mg/dL) vs. normal (0.1- <1 mg/dL)	1.1 [ 0.7–1.7]	1.6 [ 0.8–3.2]	0.8 [ 0.4–1.5]	1.1 [ 0.6–2.1]	1.1 [ 0.6–2.2]	2.3 [ 0.9–5.9]	**5.9 [ 1.9–18.5]**	**4.1 [ 1.9–9.2]**
high significant inflammation (≥10 mg/dL) vs. normal (0.1- <1 mg/dL)	1.2 [ 0.7–2.1]	2.1 [ 0.9–4.8]	1.3 [ 0.6–3.1]	1.5 [ 0.5–4.0]	1.1 [ 0.4–2.7]	2.5 [ 0.6–11.0]	2.2 [ 0.4–11.1]	**3.6 [ 1.2–10.8]**
**AST**								
elevated (>40 U/L) vs. normal (<40 U/L)	1.6 [ 0.3–7.2]	1.8 [ 0.6–5.1]	3.3 [ 0.5–21.7]	1.8 [ 0.7–4.5]	0.8 [ 0.4–1.6]	1.9 [ 0.3–10.6]	1.8 [ 0.3–11.9]	2.0 [ 0.6–7.1]
**ALT**								
elevated (>56 U/L) vs. normal (<56 U/L)	0.9 [ 0.2–3.3]	1.5 [ 0.4–5.0]	0.5 [ 0.1–3.1]	0.9 [ 0.3–3.1]	1.5 [ 0.7–3.5]	2.9 [ 0.3–25.4]	1.9 [ 0.2–23.3]	2.1 [ 0.4–12.4]
**Hypertension**								
yes vs. no	0.9 [ 0.6–1.2]	**1.6 [ 1.3–2.1]**	0.6 [ 0.3–1.1]	**2.2 [ 1.4–3.4]**	0.9 [ 0.5–1.6]	1.3 [ 0.8–2.0]	1.0 [ 0.4–2.7]	1.0 [ 0.3–3.3]

Bold = statistically significant at *p* < 0.05.

The multinomial logistic regression showed that subjects with prediabetes had higher odds of moderate NAFLD (AOR = 1.8, 96% CI = 1.1–2.8, *p* < 0.05) and severe NAFLD (AOR = 2.3, 96% CI = 1.5–3.4, *p* < 0.05) relative to the normoglycemic group after adjusting for the confounding variables. We also found that subjects with diabetes had higher odds of both moderate NAFLD (AOR = 2.3, 95% CI = 1.4–3.8, *p* < 0.05) and severe NAFLD (AOR = 4.8, 95% CI = 2.5–9.4, *p* < 0.05) relative to the normoglycemic group after adjusting for the confounding variables.

Among the total population, after adjusting for the other variables, a one unit increase in HbA1c was associated with higher odds of severe NAFLD (AOR = 1.8, 95% CI = 1.4–2.3, *p* < 0.0001). Being Mexican American, age 50 years and older, having high risk waist-to-hip ratio, being overweight or obese, and having high levels of triglycerides were associated with higher odds of both moderate and severe NAFLD compared to the other groups (*p* < 0.05). Being age 35–50 years old was also associated with higher odds of moderate NAFLD (*p* < 0.05). For the severe NAFLD stage, higher odds were additionally associated with being male, low HDL levels, and having hypertension (*p* < 0.05).

Among the populations with prediabetes and diabetes, a one unit increase in HbA1c was associated with higher odds of severe NAFLD (AOR = 2.2, 95% CI = 1.1–4.4, *p* = 0.033 and AOR = 1.5, 95% CI = 1.1–1.9, *p* = 0.003 respectively: Table 2). Among the prediabetes population, higher odds of moderate NAFLD were associated with age (≥50 years old), being overweight or obese, and not being physically active (*p* < 0.05). Severe NAFLD was associated with being overweight or obese, and borderline to high triglyceride levels (*p* < 0.05). Among the diabetes population, higher odds of moderate NAFLD were associated with age (50–64 years old), lower education, and being overweight, high levels of cholesterol and hsCRP, and borderline low levels of HDL. Severe NAFLD was associated with age 35 years and older, being overweight or obese, high levels of cholesterol, low levels of HDL, high levels of triglycerides, and hsCRP >3 mg/dL (*p* < 0.05) (Table 2). Severe NAFLD was associated with taking medications in participants with prediabetes (Supplementary Table S2; *p* < 0.05). It was also associated with taking insulin in those with diabetes, but only when not controlling for HbA1c in the regression (Supplementary Tables S2B, S2C).

#### 3.5.1 Race/ethnicity and NAFLD severity among prediabetes and diabetes populations

Among the total and normoglycemic populations, there was a racial/ethnic disparity where Mexican American had higher adjusted odds of severe NAFLD relative to Whites and Blacks had lower odds of severe NAFLD than Whites (*p* < 0.05) (Table 2). Among the prediabetes population, there was no racial/ethnic difference in the adjusted odds of moderate and severe NAFLD (*p* > 0.05). Among the diabetes population, Mexican Americans had similar adjusted odds of moderate and severe NAFLD compared to Whites, but Blacks had significantly lower adjusted odds of severe NAFLD than Whites (*p* < 0.05).

#### 3.5.2 Gender and NAFLD severity among prediabetes and diabetes populations

Among the total population and in the normoglycemic population, females had lower adjusted odds of severe NAFLD relative to males (*p* < 0.05) (Table 2). Among the prediabetes and diabetes populations, there was no gender difference in the adjusted odds of moderate and severe NAFLD (*p* > 0.05) (Table 2).

#### 3.5.3 Block regressions of the association between NAFLD and race/ethnicity and gender among prediabetes and diabetes population

Table 3 shows the results of the block regression analysis. Among those with prediabetes, after adjusting for demographics only, Mexican Americans had significantly higher odds and non-Hispanic Blacks had significantly lower odds of severe NAFLD relative to Whites. However, this model had a pseudo-R^2^ of only 0.08, indicating that it explained 8% of the variability of NAFLD. After additionally adjusting for the behavior and body composition variables, the racial/ethnic difference persisted, and the pseudo-R^2^ increased to 0.39 (*p* < 0.05). When we further adjusted for laboratory values and comorbidities, the pseudo-R^2^ increased, indicating a better fit to the data, but there was no longer a statistically significant racial/ethnic disparity.

**TABLE 3:** Hierarchical regression analysis for the association between NAFLD and race/ethnicity and gender among prediabetes and diabetes population adjusting for the confounding variables.

	Prediabetes					
Outcome = NAFLD (relative to normal/mild)	Moderate NAFLD					
**pseudo R2**	0.03	0.08	0.13	0.39	0.46	0.46
	unadjusted	demographics	Demographics + behavior	Demographics + behavior + obesity	Demographics + behavior + laboratory	Demographics + behavior + laboratory + comorbidity
**Race/Ethnicity**						
Mexican American vs. Non-Hispanic White	1.47 [0.68–3.20]	1.9 [ 0.7–5.1]	1.9 [ 0.6–5.8]	1.4 [ 0.4–4.6]	1.4 [ 0.4–4.6]	1.4 [ 0.4–4.3]
Non-Hispanic Black vs. Non-Hispanic White	0.82 [0.49–1.37]	0.8 [ 0.4–1.5]	0.8 [ 0.4–1.6]	0.7 [ 0.3–1.4]	0.8 [ 0.4–1.9]	0.9 [ 0.4–2.0]
Other Hispanic vs. Non-Hispanic White	0.85 [0.51–1.42]	1.0 [ 0.5–2.0]	1.0 [ 0.4–2.4]	0.8 [ 0.2–2.3]	0.8 [ 0.3–2.2]	0.7 [ 0.3–2.1]
Other Race vs. Non-Hispanic White	1.57 [0.86–2.85]	1.5 [ 0.7–3.2]	1.6 [ 0.7–3.2]	1.7 [ 0.8–3.6]	1.7 [ 0.7–3.7]	1.7 [ 0.7–3.7]
**Gender**						
male vs. female	0.98 [0.63–1.50]	0.9 [ 0.5–1.5]	1.0 [ 0.6–1.7]	0.9 [ 0.5–1.5]	0.9 [ 0.6–1.5]	0.9 [ 0.6–1.5]
	**PREDIABETES**					
outcome = NAFLD (relative to normal/mild)	**SEVERE NAFLD**					
**pseudo R2**	0.03	0.08	0.13	0.39	0.46	0.46
	unadjusted	demographics	Demographics + behavior	Demographics + behavior + obesity	Demographics + behavior + laboratory	Demographics + behavior + laboratory + comorbidity
**Race/Ethnicity**						
Mexican American vs. Non-Hispanic White	**1.80 [1.17–2.78]**	**2.2 [ 1.04–4.71]**	2.3 [ 0.99–5.20]	1.7 [ 0.7–4.2]	1.9 [ 0.7–4.8]	2.0 [ 0.8–5.0]
Non-Hispanic Black vs. Non-Hispanic White	**0.60 [0.38–0.94]**	**0.6 [ 0.4–0.98]**	0.6 [ 0.45–1.04]	**0.5 [ 0.3–0.9]**	0.5 [ 0.3–1.0]	0.5 [ 0.3–1.0]
Other Hispanic vs. Non-Hispanic White	**0.69 [0.50–0.96]**	0.8 [ 0.4–1.7]	0.9 [ 0.4–2.0]	0.7 [ 0.2–2.0]	0.5 [ 0.2–1.5]	0.5 [ 0.2–1.5]
Other Race vs. Non-Hispanic White	**0.59 [0.36–0.97]**	**0.5 [ 0.3–0.9]**	0.6 [ 0.3–1.0]	0.6 [ 0.3–1.2]	0.6 [ 0.3–1.2]	0.6 [ 0.3–1.2]
**Gender**						
male vs. female	0.80 [0.59–1.10]	0.8 [ 0.5–1.1]	0.7 [ 0.5–1.1]	**0.5 [ 0.3–0.9]**	0.5 [ 0.2–1.2]	0.6 [ 0.2–1.3]
	**DIABETES**					
outcome = NAFLD (relative to normal/mild)	**MODERATE NAFLD**					
**pseudo R2**	0.03	0.18	0.27	0.4	0.52	0.52
	unadjusted	demographics	Demographics + behavior	Demographics + behavior + obesity	Demographics + behavior + laboratory	Demographics + behavior + laboratory + comorbidity
**Race/Ethnicity**						
Mexican American vs. Non-Hispanic White	1.04 [0.34–3.17]	1.8 [ 0.2–21.0]	2.4 [ 0.1–45.4]	1.6 [ 0.1–33.8]	2.3 [ 0.2–30.4]	2.3 [ 0.2–30.7]
Non-Hispanic Black vs. Non-Hispanic White	0.47 [0.17–1.25]	0.3 [ 0.1–0.9]	0.3 [ 0.1–0.9]	0.3 [ 0.1–1.0]	0.4 [ 0.1–1.2]	0.4 [ 0.1–1.2]
Other Hispanic vs. Non-Hispanic White	0.39 [0.13–1.21]	0.5 [ 0.0–6.2]	0.6 [ 0.0–11.9]	0.4 [ 0.0–8.7]	0.3 [ 0.0–6.5]	0.3 [ 0.0–6.5]
Other Race vs. Non-Hispanic White	0.67 [0.34–1.34]	0.7 [ 0.3–1.7]	0.7 [ 0.3–1.5]	1.1 [ 0.4–2.8]	1.3 [ 0.5–3.7]	1.3 [ 0.5–3.8]
**Gender**						
male vs. female	1.17 [0.52–2.64]	1.3 [ 0.6–2.8]	1.2 [ 0.5–2.7]	1.0 [ 0.4–2.4]	1.6 [ 0.6–4.6]	1.6 [ 0.5–4.7]
	**DIABETES**					
outcome = NAFLD (relative to normal/mild)	**SEVERE NAFLD**					
**pseudo R2**	0.03	0.18	0.27	0.4	0.52	0.52
	unadjusted	demographics	Demographics + behavior	Demographics + behavior + obesity	Demographics + behavior + laboratory	Demographics + behavior + laboratory + comorbidity
**Race/Ethnicity**						
Mexican American vs. Non-Hispanic White	1.11 [0.46–2.73]	4.2 [ 0.6–31.9]	4.5 [ 0.3–61.9]	2.1 [ 0.2–18.7]	3.4 [ 0.8–14.7]	3.4 [ 0.8–15.2]
Non-Hispanic Black vs. Non-Hispanic White	**0.32 [0.18–0.59]**	**0.3 [ 0.2–0.5]**	**0.3 [ 0.1–0.5]**	**0.3 [ 0.1–0.6]**	**0.3 [ 0.1–0.7]**	**0.3 [ 0.1–0.8]**
Other Hispanic vs. Non-Hispanic White	0.52 [0.20–1.37]	1.6 [ 0.2–13.9]	1.6 [ 0.1–28.2]	0.9 [ 0.1–7.9]	0.8 [ 0.2–3.4]	0.8 [ 0.2–3.4]
Other Race vs. Non-Hispanic White	0.67 [0.39–1.16]	0.6 [ 0.3–1.1]	0.5 [ 0.3–1.0]	1.0 [ 0.4–2.9]	1.5 [ 0.5–4.0]	1.5 [ 0.5–4.1]
**Gender**						
male vs. female	1.04 [0.58–1.86]	1.1 [ 0.6–2.2]	1.0 [ 0.6–1.9]	0.6 [ 0.3–1.4]	1.2 [ 0.5–3.1]	1.2 [ 0.5–3.1]

Demographic variables (demographic) = gender, age, race, education, language, income ratio. Behavioral variables (behavior) = smoking, alcohol, physical activity, diet. Obesity = waist-hip ratio, BMI. Laboratory variables (laboratory) = A1c, cholesterol, HDL, CRP, triglycerides, AST, ALT. Co-morbidity (comorbidity) = hypertension.

Bold = statistically significant at *p* < 0.05

Among the population with diabetes, non-Hispanic Blacks had lower odds of severe NAFLD relative to Whites after adjusting for only the demographics. This result persisted after adjusting for each additional set of variables: behavior, body composition, laboratory, and co-morbidity variables. The model that included adjustment for all variables explained 52% of the variability of NAFLD (pseudo *R* ^2^ = 0.52; *p* < 0.05) (Table 3).

## 4 Discussion

This study examined the relationship between NAFLD severity and gender and race/ethnicity in individuals with prediabetes and diabetes among a nationally representative sample of the adults in the United States. A secondary aim was to determine the association between level of HbA1c and NAFLD severity in subjects with prediabetes and diabetes. The findings of our study indicated a high prevalence of severe NAFLD among those with prediabetes as well as diabetes, which agrees with these recent studies. In our study, we were able to determine the association between one unit increase in HbA1c and the likelihood of having moderate to severe NAFLD among the prediabetes and the diabetes populations. In addition, we were able to determine the independent variables that explained the statistically significant amount of variance in NAFLD after accounting for the other independent variables in the model.

In the overall population, we found an independent association between NAFLD and prediabetes. Subjects with prediabetes had a higher chance of developing NAFLD relative to the normoglycemic group. Similarly, participants with diabetes were also at increased odds for NAFLD relative to the normoglycemic group. Both individuals with prediabetes and those with diabetes had higher odds of severe NAFLD relative to the normoglycemic group. Our results are consistent with previous findings that both diabetes and prediabetes are risk factors for NAFLD (Lee et al., 2019; Rajput and Ahlawat, 2019; Wargny et al., 2019; Xia et al., 2019).

While the literature has documented diabetes as a major risk factor for NAFLD, recent studies have suggested that prediabetes may also be associated with NAFLD (Rajput and Ahlawat, 2019; Cuthbertson et al., 2021; Kim et al., 2022). However our study differs from these recent studies in several important ways. Several studies have examined this association in population in countries other than the United States (Song et al., 2022a; Song et al., 2022b; Kolluru et al., 2022; Succurro et al., 2022; Xu et al., 2022). While informative, the results of these studies cannot be generalized to the United States population, and typically do not allow for examination by race/ethnicity. Others have used data from United States populations, but used less accurate methods of identifying NAFLD, such as the ultrasonographic fatty liver indicator US-FLI (Ng et al., 2022). Some studies have focused on youth rather than adults (Song et al., 2022b). Some have also looked at the association between prediabetes and a metric related to NAFLD, called MAFLD, which differs in certain characteristics (Kolluru et al., 2022); however, results regarding MAFLD are not generalizable to NAFLD, given their underlying differences (Muthiah et al., 2023). Two recent studies are similar to ours in using NHANES 2017–2018 data but differ in the cutoffs used to identify NAFLD (Claypool and Patel, 2022; Kim et al., 2022). Both studies used a single cutoff of 263 dB/m as their cutoff for S1, and thus their NAFLD groups combines mild, moderate, and severe NAFLD. Using multiple cutoffs based on FibroScan**®** results patient guides, we were able to examine associations in combination with severity of NAFLD. It is also unclear from the methods of Kim et al. (2022) whether they excluded individuals with excessive alcohol intake and other hepatotoxic factors from the sample identified as having NAFLD (Kim et al., 2022). Additionally, with the exception of Claypool and Patel (2022), none of the studies mentioned investigated differences by race/ethnicity or gender, and none of the studies determined the risk associated with increases in HbA1c.

While prediabetes has long been known to be a risk factor for diabetes, more recent literature has identified additional risks related directly to having prediabetes, including cardiac conditions (Zand et al., 2018; Mai et al., 2021) and cognitive decline (Marseglia et al., 2019). The current study confirms that NAFLD needs to be included in the list of conditions that subjects with prediabetes may develop. Although there is a growing consensus that prediabetes represents a health concern, there remains debate about whether treatment should include pharmacological intervention (Shaw, 2019) in addition to lifestyle modifications. Our analyses suggested an association between medication and increased odds of severe NAFLD in participants with prediabetes, although the numbers were small and this relationship could be moderated by other factors, that were not controlled for in analyses.

In those with prediabetes and those with diabetes, we found that the odds of NAFLD increased with increasing HbA1c level, even after adjusting for all of the confounding variables. Taking insulin was associated with increased odds of severe NAFLD in those with diabetes when HbA1c was not controlled for, but not significantly associated when HbA1c was controlled for. This analysis indicates that HbA1c mediates the association between treatment and NAFLD severity. These results highlight the importance of glycemic control in reducing the odds of severe NAFLD. Recent studies in several countries have found an association between HbA1c and NAFLD in general or normoglycemic populations (Tanaka et al., 2019; Masroor and Haque, 2021; Naguib and Kassab, 2021), but most have not evaluated the association within patients with prediabetes or diabetes. One of these previous studies found that the association held after controlling for diabetes status, consistent with our findings (Masroor and Haque, 2021). Results from longitudinal studies of patients with diabetes have been mixed regarding the impact of pharmacological diabetes treatment and glycemic control on incidence and progression of NAFLD (Loosen et al., 2021; Beauchamp et al., 2022; Kramer et al., 2022; Takahashi et al., 2022). We also found that other factors such as age, race/ethnicity, gender, education, waist-hip ratio, body mass index, smoking status, and physical activity were positively associated with different stages of NAFLD among the population with prediabetes.

Although we observed a significant racial/ethnic disparity in both males and females in the normoglycemic group, we did not observe a disparity in those with prediabetes, and only in males for those with diabetes. Similarly, there was no racial/ethnic or gender difference in the high likelihood of NAFLD among those with prediabetes or with diabetes. In our hierarchical analysis, there was a racial ethnic disparity in those with prediabetes in the models controlling only for demographics. However, after controlling for behavioral variables, the difference between Mexican Americans and Whites was not observed, suggesting that one or more of the behavioral variables can account for the racial/ethnic difference. While previous literature has indicated associations between gender and race and NAFLD in the general population (Ballestri et al., 2017), studies examining predictors of NAFLD in prediabetes populations are scarce. There was no gender difference associated with incidence of NAFLD in a longitudinal study of German patients with diabetes (Loosen et al., 2021). A systematic review noted that across eight studies in “high-risk” cohorts (i.e., diabetes or obese), racial/ethnic differences were not present or were very small. This was attributed in part to underlying differences in the distributions of these risk factors between racial/ethnic groups (Rich et al., 2018). A similar argument could be made for gender differences. While the power to detect differences is reduced in the smaller subsamples of those with prediabetes and diabetes, we did not observe overly large confidence intervals that would suggest unstable estimates.

Studies have suggested that insulin resistance has a crucial role in linking NAFLD, prediabetes and diabetes (Rajput and Ahlawat, 2019). The accumulation of excess fat in the liver causes oxidative stress that can inhibit insulin signaling (Khan et al., 2019). Therefore, NAFLD can directly be related to insulin resistance leading to prediabetes and T2D. On the other hand, the excess glucose found in prediabetes or T2D reduces the ability of the liver to use glucose for energy and subsequently causing insulin resistance (Luc et al., 2019). Insulin resistance may contribute to NAFLD by increasing lipolysis of free fatty acids from adipocytes and their rerouting to the liver (Zhao et al., 2020). As a result, excess fat accumulates in the liver leading to NAFLD. Further research is needed to determine the mechanism underlying the interactions between prediabetes and NAFLD in order to uncover potential interventions.

### 4.1 Strengths

This study analyzed data from NHANES 2017–2018, which is a nationally representative sample of the non-institutionalized population of United States. In addition, FibroScan^®^, the method used for detecting NAFLD in the 2017–2018 cycle, is more sensitive than the algorithms that have been used to estimate NAFLD in the previous NHANES cycles since the 1990s.

This is the only study, to our knowledge, that examined associations of race/ethnicity and gender with NAFLD severity among prediabetes and diabetes populations. This study is also unique in that it investigated the implication of increased HbA1c in the likelihood for NAFLD among populations with prediabetes and diabetes.

### 4.2 Limitations

Limitations of our study included limited ability to assess the temporal nature of the association and make causal inference since NHANES is a cross-sectional study. For example, we were not able to assess progression of NAFLD. The FibroScan^®^ method used to identify NAFLD in this study is not as accurate as liver biopsy. However, since NAFLD is considered a relatively benign condition, biopsy is not typically implemented for its diagnosis. Additional potential predictors such as C-peptide are not currently available in NHANES (2017–2018), and low-density lipoprotein and HOMA insulin resistance were collected from a small sample. Interview data were collected by self-report, so there is a possibility of recall bias. In addition, there were no data available for some factors of interest, such as adherence to prescribed treatments. Although we controlled for major confounders, it is possible that other unknown confounders could account for the reported associations.

## 5 Conclusion

This study shows that there is an independent association between prediabetes and NAFLD in addition to the well-known association between diabetes and NAFLD. Participants with prediabetes or diabetes had a higher overall prevalence of NAFLD as well as severe NAFLD relative to the normoglycemic group. Among those with prediabetes or diabetes, increased HbA1c increased the odds for NAFLD. Longitudinal studies are needed to examine the causal relationship between prediabetes and NAFLD. Healthcare providers should screen individuals with diabetes or prediabetes for early detection of NAFLD to prevent the progression to organ damage and advocate for lifestyle interventions and possibly pharmaceutical treatment in those with prediabetes. Interventions that aim to reduce HbA1c levels among patients with prediabetes or diabetes may also reduce the risk of developing NAFLD.

## Data Availability

The data used in the study are publically available at: https://www.cdc.gov/nchs/nhanes/index.htm.
