# Supplementary material for: Racial/ethnic and gender disparity in the severity of NAFLD among people with diabetes or prediabetes
Source: Front Physiol. 2023 Feb 20;14:1076730. doi: 10.3389/fphys.2023.1076730 (PMC9986441; doi:10.3389/fphys.2023.1076730)
Supplement: Supplementary file 1 [file DataSheet1.docx]

Supplementary Material

# Supplementary Table S1. Prevalence of NAFLD severity by race/ethnicity and gender among normoglycemic, pre-diabetes, and diabetes populations

|  | **Male** | | | | | | | | |
| --- | --- | --- | --- | --- | --- | --- | --- | --- | --- |
|  | **Normoglycemic*** | | | **Prediabetes** | | | **Diabetes*** | | |
|  | **None/Mild** | **Moderate** | **Severe** | **None/Mild** | **Moderate** | **Severe** | **None/Mild** | **Moderate** | **Severe** |
|  | **# (%)** | **# (%)** | **# (%)** | **# (%)** | **# (%)** | **# (%)** | **# (%)** | **# (%)** | **# (%)** |
| **Mexican American** | 29 (35.9) | 14 (17.5) | 35 (46.5) | 21 (25.4) | 15 (19.5) | 42 (55.1) | 9 (14.5) | 8 (11.5) | 29 (73.9) |
| **Other Hispanic** | 36 (73.4) | 6 (11.3) | 12 (15.3) | 23 (42.6) | 14 (22.2) | 18 (35.2) | 6 (21.3) | 3 (7.6) | 19 (71.1) |
| **Non-Hispanic White** | 138 (65.5) | 27 (11.8) | 60 (22.7) | 97 (42.8) | 44 (17.9) | 104 (39.3) | 20 (12.9) | 16 (16.3) | 71 (70.8) |
| **Non-Hispanic Black** | 91 (81.3) | 10 (10.7) | 10 (7.9) | 84 (51.6) | 27 (19.4) | 42 (29.0) | 30 (36.0) | 12 (16.5) | 35 (47.4) |
| **Other Race** | 71 (59.6) | 19 (16.3) | 24 (24.1) | 52 (39.4) | 22 (29.8) | 45 (30.8) | 22 (26.0) | 10 (12.4) | 34 (61.6) |
|  | **Female** | | | | | | | | |
|  | **Normoglycemic*** | | | **Prediabetes** | | | **Diabetes** | | |
|  | **None/Mild** | **Moderate** | **Severe** | **None/Mild** | **Moderate** | **Severe** | **None/Mild** | **Moderate** | **Severe** |
|  | **# (%)** | **# (%)** | **# (%)** | **# (%)** | **# (%)** | **# (%)** | **# (%)** | **# (%)** | **# (%)** |
| **Mexican American** | 60 (62.0) | 14 (15.0) | 22 (23.0) | 27 (36.8) | 16 (19.8) | 40 (43.4) | 3 (11.2) | 6 (19.5) | 32 (69.2) |
| **Other Hispanic** | 32 (74.4) | 2 (5.8) | 7 (19.8) | 37 (56.2) | 12 (15.8) | 18 (28.0) | 9 (27.3) | 5 (14.0) | 14 (58.7) |
| **Non-Hispanic White** | 222 (74.0) | 45 (13.8) | 45 (12.2) | 98 (43.0) | 41 (19.6) | 78 (37.4) | 14 (15.2) | 17 (16.0) | 56 (68.7) |
| **Non-Hispanic Black** | 118 (77.1) | 22 (15.1) | 13 (7.8) | 71 (54.0) | 26 (18.2) | 38 (27.9) | 20 (27.8) | 14 (17.7) | 38 (54.5) |
| **Other Race** | 102 (75.7) | 15 (9.1) | 16 (15.2) | 63 (50.5) | 33 (31.6) | 29 (17.9) | 7 (11.7) | 9 (18.5) | 28 (69.8) |

**Supplementary Table S2A. Population characteristics and prevalence of NAFLD severity by diabetes/prediabetes treatment status**

|  | Overall | Normo-glycemic  glucose <100 mg/dL & A1c <5.7% | Prediabetes*  glucose 100-125 mg/dL or A1c 5.7-6.4% or self-report | | Diabetes  glucose > 126 mg/dL or A1c > 6.5% or self-report | | | |
| --- | --- | --- | --- | --- | --- | --- | --- | --- |
|  | Overall  # (%) | Untreated  # (%) | Treated (Oral)  # (%) | Untreated  # (%) | Insulin treatment  # (%) | Oral Hypoglycemic  # (%) | Both insulin and oral hypoglycemic  # (%) | Untreated  # (%) |
|  | n= 3190 | 1317 | 20 (1.5) | 1257 (98.5) | 43 (5.7) | 283 (47.1) | 74 (11.5) | 196 (35.8) |
| **NAFLD** |  |  |  |  |  |  |  |  |
| no/mild | 1612 (52.0) | 899 (69.5) | 3 (6.0) | 570 (44.2) | 7 (15.9) | 63 (14.7) | 23 (20.5) | 47 (20.0) |
| Moderate | 524 (16.2) | 174 (13.0) | 7 (22.4) | 243 (20.0) | 9 (14.4) | 42 (9.7) | 10 (21.8) | 39 (22.3) |
| Severe | 1054 (31.8) | 244 (17.5) | 10 (71.6) | 444 (35.8) | 27 (69.8) | 178 (75.6) | 41 (57.7) | 110 (57.8) |

**Indicates statistically significant difference at p<0.05*

**Supplementary Table S2B. Adjusted odds ratio (AOR) and 95% confidence interval for treatment and HbA1c associated with NAFLD severity (Reference population of normal/mild) in diabetes and pre-diabetes populations from NHANES 2017-18 (n=3190)**

| N=3190 | prediabetes | | diabetes | |
| --- | --- | --- | --- | --- |
|  | moderate | severe | moderate | severe |
|  | AOR* | AOR* | AOR* | AOR* |
| **Treatment** |  |  |  |  |
| Pills vs untreated | 4.2 [0.6 – 29.6] | **3.9 [1.1 – 13.7]** | 0.7 [0.3 – 1.6] | 1.7 [0.7 – 3.9] |
| Insulin vs untreated |  |  | 1.4 [0.4 – 5.9] | 2.8 [0.7 – 10.9] |
| Both medications vs untreated |  |  | 0.8 [0.1 – 4.5] | 0.4 [0.1 – 1.3] |
| **HbA1c** | 0.6 [2.9 – 1.2] | **2.1 [ 1.1 - 4.4]** | 1.1 [0.9 – 1.4] | **1.5 [1.2 – 1.9]** |

*Adjusted for all other independent variables.

Bold indicates statistical significance at p<0.05

**Supplementary Table S2C. Adjusted odds ratio (AOR) and 95% confidence interval for treatment and associated with NAFLD severity (Reference population of normal/mild) in diabetes and pre-diabetes populations from NHANES 2017-18 (n=3190), without controlling for HbA1c**

| N=3190 | prediabetes | | diabetes | |
| --- | --- | --- | --- | --- |
|  | moderate | severe | moderate | severe |
|  | AOR* | AOR* | AOR* | AOR* |
| **Treatment** |  |  |  |  |
| Pills vs untreated | 3.8 [0.5 – 27.3] | **4.4 [1.2 – 15.9]** | 0.7 [0.3 – 1.5] | 2.1 [0.9 – 4.8] |
| Insulin vs untreated |  |  | 1.6 [0.4 – 5.8] | **5.3 [1.3 – 21.8]** |
| Both medications vs untreated |  |  | 0.9 [0.2 – 4.8] | 0.7 [0.2 – 2.6] |

*Adjusted for all other independent variables except HbA1c.

Bold indicates statistical significance at p<0.05
